# Affective touch perception and longing for touch during the COVID-19 pandemic

**DOI:** 10.1038/s41598-022-07213-4

**Published:** 2022-03-10

**Authors:** Larissa L. Meijer, B. Hasenack, J. C. C. Kamps, A. Mahon, G. Titone, H. C. Dijkerman, A. Keizer

**Affiliations:** Experimental Psychology/Helmholtz Institute, Utrecht University, Heidelberglaan 1, 3584 CS Utrecht, The Netherlands

**Keywords:** Human behaviour, Sensory processing

## Abstract

Interpersonal touch and affective touch play a crucial role in social interactions and have a positive influence on mental health. The social distancing regulations implemented during the COVID-19 pandemic have reduced the ability to engage in interpersonal touch. This could cause longing for touch, and it might subsequently alter the way in which affective touch is perceived. To investigate this, we conducted an online survey and included 1982 participants, which contained questions regarding the COVID-19 regulations, longing for touch, and the perceived pleasantness of affective and non-affective touch. Results showed that participants reported feelings of longing for touch. This significantly increased with the duration and severity of the COVID-19 regulations. In addition, participants who experienced more longing for touch rated videos of affective and non-affective touch as more pleasant. Current results provide insight in the impact of sudden and prolonged COVID-19 regulations and show that increasing the duration and severity of these regulations is associated with a higher desire for touch, which is associated with increased perceived pleasantness of observing touch.

## Introduction

To contain the outbreak of the COVID-19 virus, a variety of public health measures have been implemented globally to limit physical and social interactions. One aspect of social interactions that has been particularly affected by these public health measures is the ability to engage in social touch^1^. Regulations such as quarantine and social distancing minimize social touch interactions with people outside the own household. Recently, first evidence emerged showing that the restrictions to contain the COVID-19 pandemic are linked to self-reported touch deprivation^2,3^. Longing for touch during the COVID-19 pandemic potentially has a large impact on our well-being and social life, as research shows that touch plays an integral role in social interactions^4^. It promotes the formation and maintenance of social bonds^5^, helps to convey emotions^6^, facilitates prosocial behavior^4^ and reduces feelings of ostracism^7^. Moreover, research shows that interpersonal touch is important for social support and has a positive impact on mental health. For example, holding hands with a loved one can reduce anxiety and pain^8,9^ and massages have a positive effect on psychological symptomatology^10^. In addition, interpersonal touch is associated with a higher quality of life^11^. Indeed, pre-COVID-19 research showed that touch deprivation increases stress, disrupts psychological resilience as well as coping with stressful situations, which can increase the risk of developing anxiety disorders and depression^12^. However, these previous studies have not been performed during global social distancing measures but focused on individuals who experienced touch deprivation even under unrestricted societal circumstances^13^. As such these previous findings have a limited generalizability, as during the COVID-19 pandemic a vast majority of the adult general population experienced a reduction in touch frequency, regardless of, for example, their mental health status, age or gender. It is therefore crucial to examine longing for touch in the general population during the COVID-19 pandemic, as this will further our understanding of the impact social distancing has on individuals.

Affective touch is a form of interpersonal touch that has been suggested to have beneficial effects for the individual that is being touched^14^. Affective touch refers to a gentle and slow (1–10 cm/s) stroking of the skin that is generally experienced to be very pleasant^15,16^. Gentle stroking at 1–10 cm/s (optimally at 3 cm/s) activates specific unmyelinated low-threshold mechanosensory C-fibers namely, C-tactile (CT) afferents, therefore it is also referred to as *CT-optimal touch*. CT-afferents are mostly located in the hairy skin^17^, but also see:^18^. The CT-system contains a distinct neural pathway projecting to cortical areas mostly involved in affective and emotional processing, i.e. the insula and anterior cingulate cortex (ACC)^19^. These areas have been suggested to account for the affective component of this kind of touch^12^. Besides bottom-up processing of CT-optimal touch, top-down processes, such as contextual and social factors (e.g. the intentions of the toucher, the relationship between the toucher and the individual being touched) play an important role in the appraisal of CT-optimal touch as well^12,15^. Even more so, several studies show that the top-down processes involved in CT-optimal touch are related to its beneficial effects. Several studies show that when someone is touched by their romantic partner they experience stronger pain reduction compared to being touched by an experimenter^20,21^.

In addition to contextual and social factors, other factors such as age^22^, presence of psychological disorder^23^ and exposure to touch^24^ play a top-down role in individual differences in CT-optimal touch perception. Sailer and Ackerley^24^ found that adults who reported infrequent interpersonal touch experiences perceived CT-optimal touch to be less pleasant than those who reported a higher interpersonal touch frequency. Sailer and Ackerley^24^ postulate that infrequent interpersonal touch results in a decrease in CT-optimal touch processing. As such, touch frequency appears to impact the appraisal of CT-optimal touch. This is especially of interest during the COVID-19 pandemic, as social distancing regulations might influence the amount of touch individuals receive. Changes in touch frequency as a result of COVID-19 might be associated with how CT-optimal touch is appraised and the extent to which individuals can benefit from the positive (mental health) effects of CT-optimal touch.

The first aim of the current study was to examine the link between the duration and severity of COVID-19 regulations and self-reported longing for touch in an adult community sample, while also taking certain socio-demographic factors into account, such as age^22^, gender^25^ and living conditions (e.g. living with a romantic partner or alone). We expected participants’ level of longing for touch to increase with the duration of social distancing regulations. This is in line with very recent work by Von Mohr et al.^3^ who showed that during the COVID-19 pandemic individuals crave social touch and that this craving increases with the duration of social distancing measures.

The second aim of the current study was to explore the relation between longing for touch and perceived pleasantness of touch. The perception of CT-optimal and CT non-optimal touch is typically studied using an in person paradigm in which participants are touched by the experimenter. Due to the COVID-19 pandemic it was not possible to physically interact with participants at the time of testing. We therefore included a touch paradigm in which participants observed and evaluated videos of CT-optimal and CT non-optimal touch^26,27^. Previous research shows that the mere observation of CT-optimal touch activates similar brain areas, e.g. the insula, as during the actual physical experience of CT-optimal touch^27^. Furthermore, the perceived pleasantness of observed touch also interacts with the stroking velocity^26^. Specifically, and similar to results from in person studies, participants tend to rate the observation of CT-optimal touch as more pleasant than the observation of CT non-optimal touch. Therefore, touch observation seems a reliable way to assess perception of CT-optimal and CT non-optimal touch in the current study. In addition, several factors that might influence the perception of touch were taken into account, including for example age^22^ and gender^25^. Following Sailer and Ackerley^24^ we expected that an increase in longing for touch would be related to a decrease in pleasantness ratings of CT-optimal touch videos.

## Methods

### Participants

Between April 5th and October 8th 2020, 2403 participants completed the experiment in Qualtrics. In the analyses we only included participants older than 16, who had not been diagnosed with a mental, neurological, or skin disorder and who reported that COVID-19 public health regulations were currently in effect in their country of residence. This resulted in excluding 373 participants. In addition, another 48 participants with anomalous scores on the duration of regulations variable (+ 3 SD) were excluded. This resulted in a final dataset for analyses consisting of 1982 participants. The majority of these participants were female (n = 1579), and aged between 16 and 87 (*M* = 38.53, *SD* = 15.62). All participants provided written informed consent at the start of the experiment and did not receive any form of compensation. The study was approved by the local faculty ethical review board of Utrecht University (protocol number 20-210) This study was conducted in line with the WMA Declaration of Helsinki (Fortaleza, Brazil, 2013).

Most participants lived in the Netherlands (68.1%) or Italy (11.2%) and were experiencing a lockdown at the time of testing (68.1%). This means that the government advised them to stay at home as much as possible and that social gatherings and social interactions were prohibited. Participants estimated that the COVID-19 regulations in their country of residence had been in place for an average of 42.81 days at the time of testing (*SD* = 23.92; 0–130). The majority of participants were not and had not been infected with the COVID-19 virus at the time of the experiment (75.4%), 22.7% was not sure if they had been infected. In addition, most participants were working or studying from home (59.7%) and were living with housemates with whom they had a good relationship (61.1%). A complete overview of the demographic characteristics of the sample can be found in the Table 1.

**Table 1:** Demographics of sample population.

Variables	n	%
**Gender**		
Male	396	20.0
Female	1579	79.7
Non-binary	7	0.4
**Location**		
Europe	1816	91.6
North America	33	1.7
Australia + New Zealand	9	0.5
Asia	7	0.4
South America	4	0.2
Africa	2	0.1
**Severity of regulations**		
Advice to not shake hands	34	1.7
Advice not to engage in social interactions (social distancing)	402	20.3
Lockdown	1232	62.2
Complete lockdown	314	15.8
**COVID-19**		
I am currently infected	5	0.3
I was infected in the past	29	1.5
I am/have not been infected	1494	75.4
I am unsure	449	22.7
**Living conditions**		
Living alone without housemates/pets	429	21.6
Living without housemates, but with pets	135	6.8
Living with housemates, poor relationship	129	6.5
Living with housemates, good relationship	1211	61.1
**Current employment status**		
Unemployed	302	15.2
Working or studying from home	1183	59.7
Working or studying at an external location	312	15.7
At home but unable to work/study	181	9.1

### Materials

#### Perceived pleasantness of touch videos

Participants watched two videos that depicted a forearm being stroked with a hand at a CT-optimal (3 cm/s) and CT non-optimal (30 cm/s) velocity. Each video had a duration of 10 s and the order of the videos was counterbalanced across participants (see Supplementary Materials S1 for videos). The participants completed a touch perception questionnaire after watching each video. Participants responded to five statements regarding the pleasantness of the touch: “*1. How did the videoclip make you feel? 2. How do you think the person giving the touch would rate the touch? 3. How do you think the person being touched would rate the touch? 4. How would you rate the touch? 5. How much would you like to be touched like that?*” Responses were given on a 10 point scale ranging from 0 (“*Very unpleasant*”) to 10 (“*Very pleasant*”). A mean score was subsequently calculated, with a higher score indicating that the touch observed in the video was perceived as pleasant. Cronbach's α was 0.918 for CT non-optimal velocity and 0.919 for CT-optimal velocity, demonstrating high reliability.

#### Demographic information

Information about sample characteristics (age, gender and the presence of mental/neurological disorders, current work situation) and the current COVID-related regulations (duration and severity) was obtained at the start of the study (see Supplementary Materials S1 for all demographic questions). Participants also answered a number of questions about their living conditions, including if they lived with housemates and/or pets and how they would rate the quality of their relationship with potential housemates. The latter was rated on a 10-point scale ranging from 1 (“*Very Poor*”) to 10 (“*Very Good*”). We categorized living conditions into: living without housemates and pets, living with pets, living with housemates with whom relationship quality was poor (Quality relationship < 5), and living with housemates with whom relationship quality was good (Quality relationship > 5).

#### Longing for touch

A 2-item questionnaire was used to measure longing for touch (“*Currently I would prefer to be touched by others* …” and “*Currently I would prefer to touch others* …”). Participants responded using a scale that ranged from 0 (“*Currently I would prefer to be touched less by others/to touch others less*”) to 10 (“*Currently I would prefer to be touched more by others/to touch others more*”). To calculate an average longing for touch score, the mean response was taken across these two items. Higher average scores (> 5) indicated that participants felt touch deprived. There was a high reliability between these items (Cronbach's α = 0.922).

### Data analyses

SPSS 24.0 was used to analyze the data. Data were checked for normality with a Shapiro–Wilk test and a Q–Q plot. The plots were used as additional check because of the sensitivity of the Shapiro–Wilk test to large sample sizes (Ghasemi and Zahediasl 2012). Scores on the longing for touch questionnaire were not normally distributed, as indicated by both the Shapiro–Wilk test (*p* < 0.05) and the Q-Q plots. A multiple linear regression with bootstrapping (1000 iterations) was therefore used to analyze these data. The Shapiro–Wilk test also indicated that the responses to the touch perception questionnaire were not normally distributed (*p* < 0.05). However, Q-Q plots demonstrated that these scores were approximately normally distributed. Therefore, regular linear regressions were used to analyze these variables. For all tests, the other assumptions were met. For all regressions, the VIF values for the continues variables were below 5, indicating that multicollinearity did not affect the results. Since we were dealing with a large data set we set α = 0.01 (two-tailed), unless stated otherwise.

## Results

### Longing for touch

A score of 5 on the longing for touch questionnaire would reflect a perfect balance between touch wish and touch frequency. In the current sample 82.9% (n = 1644) of the participants scored higher than 5. The average score on the longing for touch questionnaire was 7.70 (*SD* = 2.31). A one sample t-test with a test value of 5 showed that participants scored significantly higher than 5, t(1881) = 50.85, *p* < 0.001, Cohen’s D = 1.17, suggesting that participants reported to experience a longing for touch.

A multiple linear regression with bootstrapping was used to investigate the influence of the duration and severity of COVID-related regulations and four socio-demographic factors (age, gender, living conditions, current work situation) on longing for touch. The overall regression model was significant, F(13, 1796) = 14.96.24 , *p* < 0.001, R^2^ = 0.10. The regression coefficients are displayed in Table 2.

**Table 2:** Regression coefficients with longing for touch as outcome measure.

Model	B	Std. error	β	*p*
(Constant)	5.99 (5.42, 6.48)	0.28	–	0.001
Duration of regulations	0.02 (0.01, 0.02)	0.00	0.17	0.001
**Regulation severity**				
ANSH vs. complete lockdown	− 0.15 (− 1.09, 0.78)	0.44	− 0.01	0.713
Social distancing vs. complete LOCKDOWN	0.33 (− 0.61, 0.77)	0.20	0.06	0.101
Lockdown vs. complete lockdown	0.30 (− 0.01, 0.65)	0.17	0.06	0.080
Age	0.00 (− 0.01, 0.01)	0.00	0.02	0.600
**Gender**				
Non-binary vs. men	1.47 (0.46, 2.52)	0.54	0.04	0.002
Women vs. men	0.46 (0.16, 0.75)	0.16	0.08	0.0003
**Living conditions**				
Alone vs. housemates (GR)	1.02 (0.81, 1.22)	0.11	0.19	0.001
Pets vs. housemates (GR)	0.65 (0.20, 1.09)	0.22	0.07	0.003
Housemates (BR) vs. housemates (GR)	0.36 (− 0.08, 0.75)	0.20	0.04	0.071
**Current work situation**				
Unemployed vs. working externally	− 0.07 (− 0.38, 0.45)	0.17	− 0.10	0.610
Working at home vs. working externally	− 0.02 (− 0.23, 0.42)	0.12	− 0.03	0.801
Home, unable to work vs. working externally	0.08 (− 0.24, 0.72)	0.19	0.11	0.599

Multiple linear regression model (95% bias-corrected and accelerated CI reported in parentheses). Confidence intervals and standard errors based on bootstrapping samples (1000 iterations).

*ANSH* advice to not shake hands, *GR* good relationship, *PR* poor relationship.

Longing for touch was found to increase significantly with the duration of the regulations. Longing for touch was not associated with the severity of the regulations. Thus, the type of regulation that was in effect at the time of testing (advice not to shake hands, social distancing, lockdown, or complete lockdown), did not seem to modulate the level of longing for touch that was reported by participants. With respect to the socio-demographic factors, longing for touch was significantly associated with living conditions. Participants who lived alone or with pets reported to be significantly more touch deprived than participants who lived with housemates which whom they had a good relationship. Participants who lived with housemates with whom they had a bad relationship did not report higher levels of longing for touch than participants who lived with housemates with whom they had a good relationship (see Table 3). There was a significant association between longing for touch and gender, with men reporting lower levels of longing for touch than women and individuals who identified as non-binary.

**Table 3:** Mean longing for touch scores for living conditions.

Living condition	Mean	Std. deviation
Living alone	8.55	1.71
Living with pets	8.38	2.37
Living with housemates and poor relationship quality	7.71	2.26
Living with housemates and good relationship quality	7.30	2.41

### Perceived pleasantness of touch

#### Manipulation check

Prior to the main analysis, a manipulation check was conducted to determine whether participants scored higher on the touch perception questionnaire after viewing the CT-optimal touch video compared to the CT non-optimal touch video. A paired t-test showed that the CT-optimal touch video (*M* = 7.02, *SD* = 1.98) was indeed rated as significantly more pleasant than the CT non-optimal touch video (*M* = 3.37, *SD* = 1.96), t(1947) = − 64.94, *p* < 0.001.

#### Main analysis

Two multiple linear regressions were conducted with perception of CT-optimal and CT non-optimal touch as respective outcome measures. The level of longing for touch, the severity and the duration of COVID-related regulations were included as between-subjects factors. Gender and age were also included as predictors in the model, to control for the potential influence of these variables. The first model was a significant predictor for the perceived pleasantness of the CT-optimal touch video, F(8, 1858) = 42.59 *p* < 0.001, R^2^ = 0.16. The regression coefficients can be found in Table 4.

**Table 4:** Regression coefficients with perceived pleasantness of CT-optimal touch observation as outcome measure.

Model	B	Std. error	β	t	*p*
(Constant)	4.59 (4.19, 4.99)	0.20	–	22.77	< 0.001
Longing for touch	0.28 (0.24, 0.31)	0.02	0.33	14.82	< 0.001
Duration of regulations	0.00 (− 0.00, 0.01)	0.00	0.010	0.35	0.730
**Regulation severity**					
ANSH vs. complete lockdown	− 0.49 (− 1.17, 0.18)	0.34	− 0.03	− 1.44	0.150
Social distancing vs. complete lockdown	− 0.54 (− 0.84, − 0.24)	0.15	− 0.11	− 3.52	< 0.001
Lockdown vs. complete lockdown	− 0.61 (− 0.84, − 0.37)	0.12	− 0.15	− 5.12	< 0.001
Age	0.02 (0.02, 0.03)	0.00	0.18	8.09	< 0.001
**Gender**					
Non-binary vs. men	− 0.30 (− 1.63, 1.04)	0.68	− 0.01	− 0.44	0.661
Women vs. men	− 0.02 (− 0.23, 0.19)	0.11	− 0.04	− 0.19	0.848

Multiple linear regression model (95% CI in parentheses).

*ANSH* advice to not shake hands.

**Figure 1:**
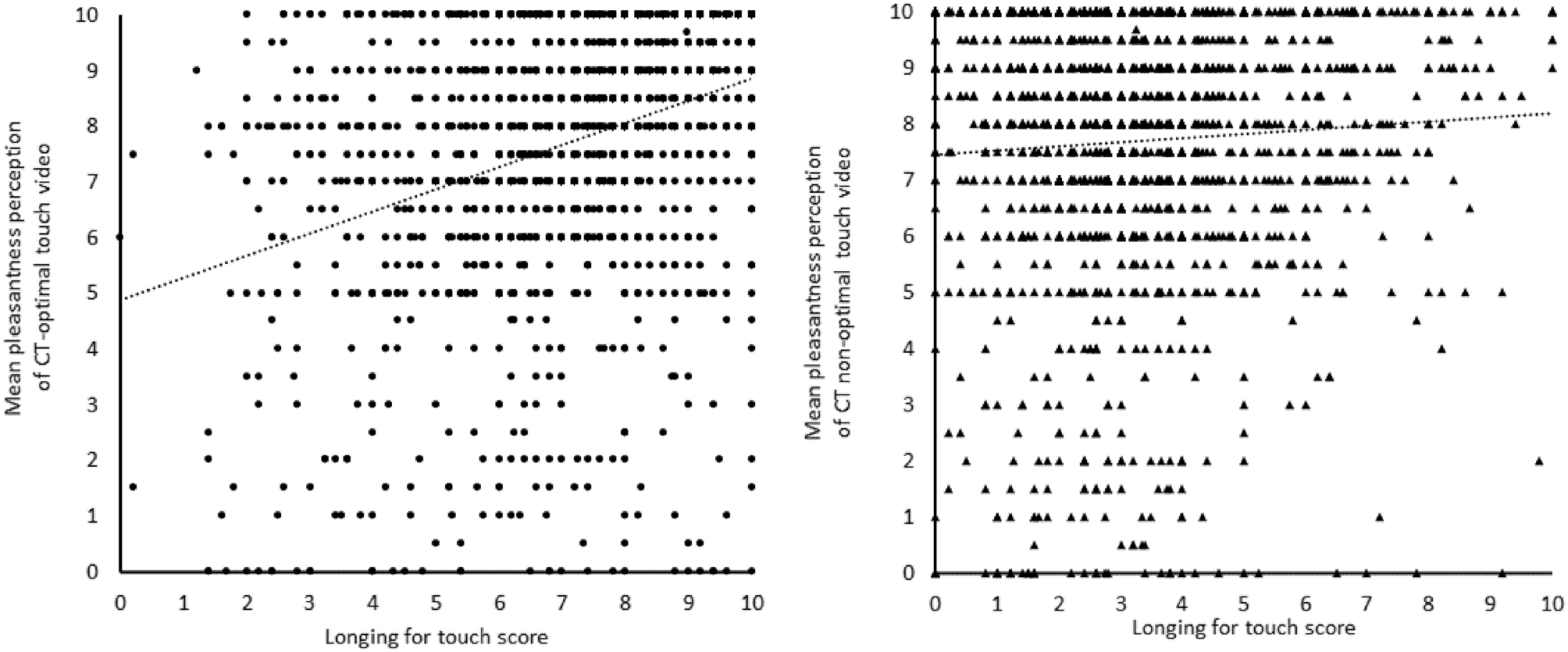
The relation between longing for touch and pleasantness perception of CT-optimal and CT non-optimal videos. The left panel depicts the individual data points and relation between longing for touch and pleasantness perception of the CT-optimal video. The right panel depicts the individual data points and relation between longing for touch and pleasantness perception of the CT non-optimal video.

The perceived pleasantness of the CT-optimal touch video significantly increased as the level of longing for touch (see Fig. 1) and age increased. Participants in complete lockdown also perceived the CT-optimal touch video to be significantly more pleasant than those who were under social distancing measures and those in lockdown. There was no difference in pleasantness ratings of CT-optimal touch videos between participants who were advised not to shake hands and participants who were in complete lockdown. The perceived pleasantness of the CT-optimal touch video was not significantly associated with the duration of regulations, nor gender.

The second model significantly predicted the perceived pleasantness of the CT non-optimal touch video, F(8, 1852) = 6.84, *p* < 0.001, R^2^ = 0.03. The regression coefficients can be found in Table 5.

**Table 5:** Regression coefficients wit perceived pleasantness of CT non-optimal touch observation as outcome measure.

Model	B	Std. error	β	t	*P*
(Constant)	3.62 (3.19, 4.05)	0.22	–	16.41	< 0.001
Longing for touch	0.08 (0.04, 0.12)	0.02	0.10	4.09	< 0.001
Duration of regulations	− 0.00 (− 0.01, 0.00)	0.00	− 0.00	0.12	0.100
**Regulation severity**					
ANSH vs. complete lockdown	− 0.38 (− 1.13, 0.36)	0.38	− 0.03	− 1.01	0.315
Social distancing vs. complete lockdown	− 0.77 (− 1.10, − 0.45)	0.17	− 0.16	− 4.64	< 0.001
Lockdown vs. complete lockdown	− 0.50 (− 0.76, − 0.25)	0.13	− 0.12	− 3.89	< 0.001
Age	> 0.001 (− 0.00, 0.00)	0.00	0.00	0.02	0.986
**Gender**					
Non-binary vs. men	− 0.95 (− 2.41, 0.51)	0.13	− 0.03	− 1.28	0.201
Women vs. men	− 0.31 (− 0.54, − 0.10)	0.12	− 0.06	− 2.72	0.007

Multiple linear regression model (95% CI in parentheses).

*ANSH* advice to not shake hands.

Pleasantness scores for the CT non-optimal touch video were significantly and positively associated with levels of longing for touch. In addition, participants in complete lockdown perceived the CT non-optimal touch video to be significantly more pleasant than those who were socially distancing and those in lockdown. There was no difference in pleasantness ratings of CT non-optimal touch videos between participants who were advised not to shake hands and participants who were in complete lockdown. Women perceived the CT non-optimal touch video to be less pleasant than men. Individuals who identified as non-binary did not differ from men in terms of pleasantness ratings for the CT non-optimal video. There was no significant association between pleasantness ratings of the CT non-optimal video and the duration of the regulations, and age.

## Discussion

Interpersonal touch has been found to play an important role in social bonding and has a positive influence on mental health^8^^4^. During the COVID-19 pandemic, several regulations have been in effect to prevent the virus from spreading. Restrictions such as quarantine, lockdown, and social distancing have decreased the frequency of touch interactions outside the own household, which could result in feelings of touch deprivation^3^. Previous work on touch deprivation shows a link between touch deprivation and altered perceived pleasantness of CT-optimal touch^13,24^. However, these studies have not been conducted in a large community sample experiencing restrictions with respect to social interactions. The aim of the current study was therefore to investigate if individuals report feelings of longing for touch under COVID-19 regulations, which factors contribute to longing for touch and if the level of longing for touch is associated with pleasantness perception of touch.

To assess if participants felt touch deprived, we asked them to indicate on a ten-point scale whether they would like to receive less touch/touch others less (0) or receive more touch/touch others more (10). On average participants scored 7.70, implying that participants longed for touch at the time of testing, as only a score of 5 would reflect a perfect balance between touch wish and touch frequency. Our findings are in accordance with the recent findings by Von Mohr et al.^3^, who also report a craving for touch during COVID-19 in their sample, especially with respect to professional and friendly touch, and to a lesser extent with respect to intimate touch. As we do not have longing for touch scores of our sample before the COVID-19 pandemic, we cannot draw any conclusions with respect to how the pandemic has impacted longing for touch scores. In other words, at this point we do not know whether the longing for touch score of 7.70 that we report here reflects a (significant) increase in feelings of longing for touch. However, a pre COVID-19 study of Beßler, Bendas, Sailer, and Croy^28^ showed that over 70% of the healthy individuals in their sample reported that they experienced a longing for touch. So, it appears that even in a society in which there are no restrictions with respect to social interactions, individuals experience a discrepancy between the amount of touch they would want to receive and the amount of touch they actually receive. Interestingly, in our sample 82.9% of the participants reported that experienced a longing for touch. Although a direct statistical comparison between our post COVID-19 longing for touch scores and the pre COVID-19 longing for touch scores reported by Beßler et al.^28^ is not possible, we cautiously suggest that the restrictions following from the COVID-19 pandemic have had a negative impact on the level of longing for touch reported in the community.

In addition, we investigated which sociodemographic variables were associated with variability in longing for touch scores. The results showed that higher levels of longing for touch were linked to a longer duration of COVID-19 regulations. This is in line with the recent work of Von Mohr et al.^3^ who also found that longing for touch is associated with the duration of COVID-19 and social distancing regulations. We further extend the findings by Von Mohr et al.^3^ by showing that not only the duration of the COVID-19 regulations is associated with higher levels of longing for touch but also the living situation of the participants. Participants who lived alone or who lived with pets reported higher levels of longing for touch than participants who lived with housemates with whom they had a good relationship. These findings are in accordance with recent work by^2^, who also showed that living situation correlated with longing for touch. We did, however, not observe a difference in longing for touch levels between participants who lived with housemates with whom they had a bad relationship and participants who lived with housemates with whom they had a good relationship. Participants living with housemates might have had more opportunities to engage in human touch interactions compared to participants living alone or with pets. Nevertheless, participants living with housemates still reported to feel touch deprived at time of testing. Even participants living with housemates with whom they had a good relationship still had a longing for touch score of 7.30 (on a ten-point scale). This might be explained by findings of Von Mohr et al.^3^, who reported that under COVID-19 regulations individuals seem to especially crave friendly *and* professional touch. This highlights the importance of touch interactions with a variety of touch partners, e.g. significant others, but also friends or colleagues, for maintaining a satisfying balance between the need for touch and touch frequency. Participants who lived with housemates might thus still have craved touch interactions with individuals outside their household.

It should be noted that our regression model explained almost 10% of the variation in longing for touch. We included a limited amount of variables and it is clear that (multiple) other factors might also impact longing for touch. Future studies are needed to construct a complete and coherent overview of which variables determine how much one craves touch.

The second aim of our study was to explore the relation between longing for touch and the perceived pleasantness of observing touch during COVID-19. Participants watched short touch videoclips and rated the pleasantness of these videoclips. We specifically investigated which factors were associated with the appraisal of touch observation. We found a positive association between longing for touch and pleasantness ratings for both CT-optimal and CT non-optimal touch videoclips, indicating that participants who longed for touch more reported higher levels of pleasantness when observing touch. This might be explained by top-down mechanisms such as social and contextual factors that have been found to be involved in the perception of CT-optimal touch^12,15^. It could be that the restrictions in social interaction increased our desire to be touched which led to a higher appraisal of touch. In addition, appraisal of CT-optimal touch is also linked to activation of brain areas involved in the reward system^29^, it could be that in our study increased pleasantness ratings of touch reflect an increased activation of the reward system. A parallel can be drawn here with work focusing on the positive relation between food deprivation and the subjective appraisal of high-calorie foods^30^. Similarly, craving touch might make the observation of touch more appealing. This explanation fits with our finding that not only CT-optimal touch videoclips were rated as more pleasant when longing for touch increased, but that the same pattern was observed for videoclips depicting CT non-optimal touch. It should however be noted that the explained variance for pleasantness ratings of CT-optimal touch videos was 16%, while the explained variance was only 0.3% for CT non-optimal touch videos. Nevertheless, a potentially interesting hypothesis following from this line of reasoning is that even forms of touch that do not necessarily activate CT-afferents may become more desirable when there are fewer opportunities to receive touch. To further explore this, future studies could focus on investigating how the perception of specific types of physical interactions (i.e. a handshake or an accidental brush) changes as longing for touch increases.

Our findings are in contrast with previous work that indicated that touch deprived participants experienced CT-optimal touch as less pleasant than participants who were not touch deprived^24^. Sailer and Ackerley^24^ suggest that infrequent CT-optimal touch experiences shape the interpretation and hedonic evaluation of those experiences. The contrasting findings between our study and that of Sailer and Ackerley^24^ might be explained by the fact that Sailer and Ackerley^24^ conducted an in person study, in which participants were actually touched, while we conducted a study in which participants observed videos depicting touch. Although observation of touch and being physically touched are different, earlier studies focusing on touch observation did show that observing CT-optimal touch activates the same brains regions as being physically touched^27^. Moreover, when observing touch videos, participants rated CT-optimal touch videos as more pleasant than CT non-optimal videos^26^, similar to what is typically found in in person touch experiments. This suggests that top-down mechanisms also play an important role in the perceived pleasantness of CT-optimal touch. Future studies in which the interplay between longing for touch and pleasantness perception of both observed and physical touch is investigated could shed further light on this issue.

An alternative explanation for the contrasting findings between our study and that of Sailer and Ackerley^24^ might be found in the different social circumstances under which the studies were conducted. The study by Sailer and Ackerley^24^ was conducted in a society with no restrictions on social interactions. As such their participants were potentially touch deprived as a result of, for example, a limited social network or limited amount of touch partners. Indeed Sailer and Ackerley^24^ report that the majority of their touch deprived group did not have a partner and/or child(ren), while the majority of their control group did. In contrast, in our study participants’ social network did not necessarily change, but the opportunity to engage in touch interactions with their social network *did *change as a result of the pandemic. As such, it could be that in the study of Sailer and Ackerley^24^ different social and contextual factors, such as the setting in which the study took place influenced the appraisal of CT-optimal touch.

We furthermore found a link between age and pleasantness perception of CT-optimal touch observation. This is in line with previous observations showing that pleasantness ratings for CT-optimal touch increase with age^22^. We also found that participants in a complete lockdown perceived both CT-optimal and CT non-optimal touch videos to be more pleasant than those who were socially distancing and those who were in lockdown. Although this effect is not necessarily unexpected, it might not be entirely driven by the level of restrictions, as we also observed that pleasantness ratings of both CT-optimal and CT non-optimal touch videos were not different for those who were advised not to shake hands and those in complete lockdown. This could however be due to an unbalanced number of participants across each category of regulation severity. Less than 2% of our sample indicated that in their country of residence the only restriction in effect was an advice not to shake hands. A more likely explanation for higher pleasantness ratings of touch videos by those in a complete lockdown may be related to cultural differences. The majority of the participants in a complete lockdown lived in Italy. This is considered to be a high-contact culture in which, as recent research suggests, CT-optimal touch is more prevalent^31^. We therefore speculate that perhaps a combination of more severe COVID-19 regulation and living in a high contact culture both have resulted in higher levels of observed touch pleasantness. It should however be noted that in our study we only asked participants to indicate which restrictions were in place at the time of participation, but we did not ask participants to indicate whether they indeed adhered to these regulations. The results with respect to pleasantness perception of touch videos and regulation severity should thus be interpreted with caution.

Even though pleasantness perception of touch videos appeared to be predicted by regulation severity, we did not find an link between regulation severity and longing for touch. Thus, regulation severity seemed to predict how pleasant participants rated the touch videos, but it did not predict how much participants longed for actual touch. This is a remarkable findings for which we do not have a clear explanation at this point.

Another limitation of the current study is that we did not take different types of social touch and interaction partners into account, which could have provided us with more information regarding the way in which individuals feel touch deprived. Von Mohr et al.^3^ did distinguish between professional, friendly, and intimate touch and showed that the difference in touch frequency pre- and post COVID-19 was largest for friendly touch. However, the participants also reported that they craved intimate touch most and that this increased with duration of the COVID-19 regulations. These findings highlight the complexity of our need for touch and how this may impact feelings of touch deprivation.

To conclude, our results demonstrate that an increased duration of COVID-19 regulation is associated with higher levels of longing for touch in the community. It seems that individuals who live alone or who live with pets suffer from the higher levels of longing for touch, compared to individuals who live with housemates. In addition, longing for touch appears to be related to touch pleasantness perception. We found that higher levels of longing for touch were linked to a more pleasant perception of videoclips showing both CT-optimal and CT non-optimal touch. Thus, individuals who long for touch more find it more pleasant to watch videoclips showing interpersonal touch. We suggest that fewer opportunities to engage in touch may potentially increase the hedonic value ascribed to touch stimuli, similar to an increased liking of high calorie foods when food deprived. Our study contributes to the understanding of the factors that are associated with longing for touch and how this links to touch perception in (healthy) adults. By doing so, this study also provides new insights into the wider consequences of COVID-19-related public health measures.

## Supplementary Information

Supplementary Information.
