# Supplementary material for: Affective touch perception and longing for touch during the COVID-19 pandemic
Source: Sci Rep. 2022 Mar 10;12:3887. doi: 10.1038/s41598-022-07213-4 (PMC8913618; doi:10.1038/s41598-022-07213-4)
Supplement: Supplementary file 1 — Supplementary Information. [file 41598_2022_7213_MOESM1_ESM.pdf]

Supplementary Materials

Touch Videos

Video 1: https://www.youtube.com/watch?v=_mw1Lkmc1s8&ab_channel=anoukkeizer *Video, with a duration of 10 seconds, depicting a forearm being stroked by a hand with a CT-optimal (3 cm/s) velocity.*

Video 2: https://www.youtube.com/watch?v=yPLpx_nvTHk&ab_channel=anoukkeizer *Video, with a duration of 10 seconds, depicting a forearm being stroked by a hand with a* CT non-optimal (30 cm/s) *velocity.*

Sociodemographic questionnaire

1. In which country are you living right now?

2. In your country are there currently regulations in effect or was there regulations in effect in the past in relation to social interactions? [There are regulations currently in effect; There were regulations in effect in the past; There are no regulations in effect now or in the past]

3. What is the highest level of regulations that right now applies in your country with respect to social interaction? [Advice not to shake hands ; Advice not to engage in social interactions (social distancing); Lockdown (e.g. advice to stay at home unless you need to go out, prohibition of social gatherings and interactions, fines in order for those who do not adhere to regulation); Complete lockdown (e.g. prohibited to leave the house without a clear purpose (i.e. groceries/work), stay in a close radius of your house, most stores and businesses closed)]

4. What was the highest level of regulations that applied in your country in the past with respect to social interaction? [Advice not to shake hands ; Advice not to engage in social interactions (social distancing); Lockdown (e.g. advice to stay at home unless you need to go out, prohibition of social gatherings and interactions, fines in order for those who do not adhere to regulation); Complete lockdown (e.g. prohibited to leave the house without a clear purpose (i.e. groceries/work), stay in a close radius of your house, most stores and businesses closed)]

5. How long have you been without regulations? [Days….]

6. For how long were these regulations with respect to social interactions in effect in your country?[Days….]

7. For how long have these regulations with respect to social interactions been in effect in your country? [Days….]

8. Have the regulations been scaled down in your country? [Yes; No]

9. For how long have the regulations been scaled down? [Days….]

10. What was the highest level of regulations that applied in your country in the past with respect to social interaction before they were scaled down? [Advice not to shake hands ; Advice not to engage in social interactions (social distancing); Lockdown (e.g. advice to stay at home unless you need to go out, prohibition of social gatherings and interactions, fines in order for those who do not adhere to regulation); Complete lockdown (e.g. prohibited to leave the house without a clear purpose (i.e. groceries/work), stay in a close radius of your house, most stores and businesses closed)]

11. Are you or have you been infected with COVID-19 virus? [I am currently infected; I was infected in the past; I am not and have not been infected; Unsure]

12. How old are you? [Years…]

13. What is your gender? [Male; Female; Non-binary]

14. Do you have a romantic partner? [Yes; No]

15. Are you living together with your romantic partner? [Yes; No]

16. Are you living together with your family? [Yes; No]

17. Are any of your children living at home with you? [Yes; No]

18. Are any pets living with you at home? [Yes; No]

19. With how many people do you share your house? [People…]

20. How would you rate the quality of your relationship with your housemate(s)? [0 Very poor – 10 Very good]

21. Are you currently afflicted with a neurological disorder or skin condition that affects your touch perception? [Yes; No; Prefer not to say]

22. Are you currently diagnosed with a mental disorder? [Yes; No; Prefer not to say]

23. Choose the option that best describes your current occupation [Unemployed (e.g. no current paid job, stay at home parent, retired); At home but unable to work/study; Working/studying from home; Working/studying at external location]
